# Genetic Architecture of Cognitive Resilience in Alzheimer’s Disease: Mechanisms, Pathways, and Therapeutic Implications

**DOI:** 10.3390/neurolint18030050

**Published:** 2026-03-03

**Authors:** Gabriel Burdman, Juliet Akkaoui, Natalia Colon, Andres Perez, Madepalli K. Lakshmana

**Affiliations:** 1 Department of Cellular and Molecular Medicine, Herbert Wertheim College of Medicine, Florida International University, Miami, FL 33199, USA; gburd004@fiu.edu (G.B.); jakka001@fiu.edu (J.A.); ncolo037@fiu.edu (N.C.); apere1398@fiu.edu (A.P.); 2 College of Arts, Sciences and Education, Florida International University, Miami, FL 33199, USA

**Keywords:** Alzheimer’s disease, cognitive resilience, clinicopathologic dissociation, genetic modifiers, amyloid-β, tau, synaptic integrity, microglia, lipid metabolism, APOE

## Abstract

**Background/Objectives**: Alzheimer’s disease (AD) is defined by amyloid-β plaques and tau neurofibrillary tangles and is typically associated with progressive cognitive decline. However, a substantial subset of individuals remains cognitively intact despite intermediate-to-high AD pathology, a phenomenon termed cognitive resilience. This review aims to synthesize genetic variants and biological pathways associated with preserved cognition in the presence of AD neuropathology. **Methods**: We performed a narrative thematic synthesis of human genetic studies (GWAS, sequencing, biomarker-informed cohorts) and extreme resilience case reports. Variants were prioritized by replication, mechanistic plausibility, and relevance to clinicopathologic dissociation, and were organized by shared biological pathways. When applicable, cognitive resilience was operationalized using residual-based approaches modeling cognitive performance after adjustment for neuropathological burden, age, sex, and education or cognitive reserve proxies reported by each cohort. **Results**: Recurrent resilience-associated variants include *APOE* ε2, *APOE3*-Christchurch, *RELN*-COLBOS, *ATP8B1*, *RAB10*, *PLCG2*, *PICALM*, *CLU*, *FN1*, and synapse-linked markers such as *NPTX2*. These variants converge on lipid metabolism, synaptic function and neuroplasticity, tau regulation and proteostasis, immune and inflammatory signaling, vascular/BBB resilience, and RNA regulation. **Conclusions**: Genetic determinants of cognitive resilience highlight mechanisms that preserve neural integrity independent of pathological load. Targeting resilience pathways may enable precision therapies designed to maintain cognitive function in AD.

## 1. Introduction

Alzheimer’s disease (AD) has long been defined by a deterministic biological framework: the amyloid cascade hypothesis. This model posits a linear progression in which the accumulation of amyloid-beta (Aβ) plaques triggers the spread of hyperphosphorylated tau neurofibrillary tangles, precipitating synaptic disintegration, neuronal death, and inevitable cognitive decline. For decades, this paradigm has driven therapeutic development under the assumption that substantial neuropathology dictates clinical dementia. However, a growing body of clinicopathologic evidence challenges this monolithic view. Autopsy studies, particularly from large longitudinal cohorts such as the Religious Orders Study and the Rush Memory and Aging Project (ROSMAP), consistently reveal that approximately 30 to 50 percent of older adults who meet full neuropathological criteria for AD, including intermediate to high Braak stages of neurofibrillary involvement and significant amyloid burden, remain cognitively unimpaired until death [1].

This phenomenon, termed clinicopathologic dissociation, implies that the brain possesses intrinsic defense mechanisms capable of buffering the toxic effects of proteopathy. These mechanisms allow neurnetworks to maintain functional integrity despite the presence of molecular insults that would typically cause catastrophic failure. This capacity is defined as cognitive resilience. Unraveling the genetic and molecular architecture of resilience represents a frontier in neurodegenerative research, offering a complementary strategy to disease-modifying therapies. Rather than solely attempting to eradicate pathology, a strategy that has thus far proven elusive, therapeutic efforts may instead focus on fortifying the brain’s ability to withstand pathological stress.

### 1.1. Conceptual Framework: Resistance, Resilience, and Reserve

To rigorously investigate the genetics of this phenomenon, the field has moved toward precise definitions that distinguish between avoiding pathology and coping with it. As delineated by Arenaza-Urquijo and Vemuri and further expanded by Stern and Montine, the following three distinct but overlapping concepts must be clarified [2].

#### 1.1.1. Alzheimer’s Disease Resistance

Resistance refers to biological processes that prevent or delay the initial accumulation of primary AD pathologies. A resistant individual exhibits low or absent levels of amyloid and tau despite possessing risk factors such as advanced age or the *APOE* ε4 allele. For example, the APP A673T mutation reduces cleavage of amyloid precursor protein by beta-secretase, thereby lowering Aβ production. This represents a mechanism of resistance rather than resilience, as the pathological stressor is avoided entirely [3].

#### 1.1.2. Cognitive Resilience

Cognitive resilience describes the capacity of the brain to maintain normal cognitive function despite the presence of significant AD pathology. A resilient individual harbors high levels of plaques and tangles, often indistinguishable from those observed in individuals with clinical dementia, yet demonstrate preserved cognition. This implies the presence of active buffering mechanisms, including enhanced synaptic plasticity, metabolic flexibility, and attenuation of maladaptive neuroinflammatory responses, which collectively uncouple proteinopathy from neurotoxicity [4].

#### 1.1.3. Cognitive Reserve

Cognitive reserve is often used interchangeably with resilience, but typically refers to the passive or active capacity built through life experiences such as education, occupational complexity, and bilingualism that enables individuals to tolerate pathology. Reserve is commonly measured using indirect proxies, whereas resilience reflects the physiological manifestation of this tolerance at the molecular and cellular levels [5].

This report focuses primarily on the genetic architecture of cognitive resilience. Unlike lifestyle-driven contributors to reserve, genetic resilience factors provide direct molecular entry points for therapeutic intervention. By identifying variants that naturally decouple pathology from dementia, effectively serving as experiments of nature, it becomes possible to pinpoint specific biological pathways that protect the brain.

### 1.2. The Genetic Landscape: From Common Variants to Extreme Phenotypes

The genetic study of Alzheimer’s disease has historically emphasized risk-conferring variants such as *APOE* ε4, TREM2, and SORL1. In contrast, recent genome-wide association studies of resilience, often operationalized as residual cognitive performance after adjusting for neuropathological burden, along with deep sequencing of rare extreme phenotypes, have identified a distinct set of loci associated with protection rather than risk. Notably, these resilience-associated variants do not necessarily reduce amyloid burden but instead modulate the cellular response to amyloid and tau pathology.

These genetic factors can be organized into functional pillars based on their dominant biological mechanisms:

- Lipid metabolism and ApoE–heparan sulfate proteoglycan interactions, which influence tau propagation and membrane repair;
- Synaptic function and neuroplasticity, which stabilize synaptic transmission and excitatory-inhibitory balance under proteotoxic stress;
- Endosomal-lysosomal trafficking, which ensures efficient routing and degradation of toxic substrates and prevents intracellular congestion;
- Innate immunity and microglial state, which tune immune responses toward clearance and tissue repair rather than chronic neurotoxicity;
- Vascular integrity and blood–brain barrier function, which maintain metabolic support and clearance pathways essential for neuronal survival.

The sections that follow provide a detailed analysis of the specific genes, variants, and mechanisms operating within each of these pillars, supported by evidence from human genetics, transcriptomics, and experimental model systems.

## 2. Materials and Methods

A structured literature search was conducted using the PubMed database to identify studies investigating genetic modifiers of cognitive resilience in AD. Search terms included combinations of AD, cognitive resilience, clinicopathologic dissociation, protective genetic variants, amyloid, tau, microglia, synaptic integrity, and blood–brain barrier. Since there are no general guidelines for the selection and classification of resilient factors, we went to through a comprehensive list of case reports to explore the influence of common genetic variants that influenced the cognitive outcomes. Most studies used standard cognitive measures but the confounding factors such as socioeconomic status, age, education level, and physical health factors like body mass index (BMI) were not always reported. The search was limited to peer-reviewed English-language articles published between January 2020 and March 2025. Eligible studies included human genetic analyses (GWAS, whole-exome or whole-genome sequencing), biomarker-informed cohort studies, and meta-analyses. Extreme-phenotype case reports were included when supported by mechanistic or translational evidence, while animal and in vitro studies were considered only when directly linked to human genetic findings.

To systematically evaluate and prioritize genetic contributors, genes were classified using a predefined four-tier evidence framework (Tiers A–D; Table 1). Genes were assigned to tiers based on phenotype relevance, strength of human evidence, degree of replication, and mechanistic support. This framework ensured consistent interpretation across heterogeneous study designs and enabled a clear distinction between resilience mechanisms and pathology-reducing effects.

To ensure phenotypic rigor, resilience was operationalized as preserved cognitive performance relative to pathological burden, assessed using neuropathology, cerebrospinal fluid biomarkers, or amyloid and tau PET imaging when available. Genes were classified as resistance modifiers when primary evidence indicated reduced pathology formation rather than preserved cognition at matched pathology levels. Biomarker-linked genes were included only when supported by replicated human genetic associations or consistent resilience-related expression patterns.

## 3. Results

Protective genetic factors associated with cognitive resilience converge on a limited number of biological pathways that buffer neuronal systems from amyloid/tau toxicity, maintain synaptic integrity, optimize glial state transitions, and preserve clearance routes across vascular and endolysosomal systems. A summary of key variants, pathway assignments, evidence tiers, and representative studies is provided in Table 2.

### 3.1. Lipid Metabolism and Amyloid Processing

Variants in this domain influence cholesterol transport, lipoprotein lipidation, membrane composition, and protein aggregation dynamics that shape Aβ deposition and downstream tau injury.

#### 3.1.1. *APOE* ε2 (Tier A)

Normal function: Apolipoprotein E (APOE) supports neurite outgrowth and membrane repair, and shapes synaptic remodeling through lipid delivery and receptor signaling (e.g., LDLR/LRP1). APOE also influences amyloid-β (Aβ) aggregation and clearance, microglial responses to plaques, and downstream vulnerability to tau-mediated neurodegeneration [25,26].

Resilience association: The *APOE* ε2 allele (rs7412; Arg158Cys) is the most robust common genetic factor associated with protection from late-onset AD, demonstrating reduced AD risk, delayed symptom onset, and slower cognitive decline compared with ε3 and especially ε4 across multiple large cohorts. Importantly, ε2 is also enriched among individuals who maintain cognition longer than expected given amyloid and/or tau burden, consistent with a resilience phenotype rather than simple absence of pathology [27,28,29].

Proposed mechanism: *APOE* ε2 differs from ε3/ε4 in receptor binding and lipidation behavior, producing a CNS environment more supportive of synaptic maintenance under proteotoxic stress. First, ε2 is typically more lipidated (ABCA1-dependent), which improves cholesterol efflux from astrocytes and delivery to neurons, strengthening membrane repair capacity, dendritic spine stability, and synaptogenesis processes essential for maintaining cognitive function when amyloid and tau are present. Second, ε2 exhibits lower propensity for pathological self-association and may form less neurotoxic ApoE–Aβ assemblies, facilitating clearance of soluble Aβ species and reducing oligomer-mediated synaptic dysfunction. Third, APOE isoforms modulate microglial activation states; ε2-associated lipid handling is linked to less maladaptive inflammatory signaling and improved plaque containment, which may indirectly limit tau phosphorylation and propagation [25,26,30].

Therapeutic implications: *APOE* ε2 biology supports resilience-oriented interventions that strengthen lipidation and neuronal repair programs rather than solely targeting amyloid removal. Candidate strategies include enhancing APOE lipidation (e.g., ABCA1/LXR pathway modulation), shifting APOE toward ε2-like functional properties, or allele-informed delivery approaches that increase neuroprotective lipid transport while minimizing inflammatory activation. Such approaches may be most effective when combined with tau- or synapse-targeted therapies to preserve cognition despite ongoing pathology [28].

#### 3.1.2. *APOE3*-Christchurch (R136S) (Tier A)

Normal function: Beyond lipid shuttling, APOE interacts with cell-surface receptors and heparan sulfate proteoglycans (HSPGs), facilitating the cellular uptake of extracellular ligands, including amyloid-β and tau species, and modulating glial responses to protein aggregates. These interactions place APOE at a critical interface between extracellular proteopathy and intracellular neurodegenerative cascades [26,31].

Resilience association: In an autosomal-dominant AD, homozygosity for the rare *APOE3*-Christchurch (R136S) variant was identified in a PSEN1 E280A mutation carrier who remained cognitively unimpaired until her seventh decade of life—nearly three decades beyond the expected age of dementia onset. Remarkably, neuroimaging and postmortem analyses revealed an exceptionally high amyloid burden comparable to or exceeding that of symptomatic AD cases, while tau pathology remained largely restricted to medial temporal regions with minimal neocortical spread. This striking clinicopathologic dissociation provides a powerful proof-of-concept of extreme cognitive resilience. However, because *APOE3*-Christchurch has been observed in a single extended pedigree, these findings should be interpreted as hypothesis-generating rather than broadly representative of resilience mechanisms in the general population [7].

Proposed mechanism: The R136S substitution is located within the APOE heparin-binding domain (residues 136–150), a positively charged region required for high-affinity binding to neuronal HSPGs. This mutation markedly reduces APOE–HSPG interactions, disrupting a key co-receptor pathway that mediates neuronal uptake of extracellular tau seeds. Impaired HSPG-dependent internalization limits trans-synaptic tau propagation, effectively uncoupling amyloid accumulation from downstream tau spread and neurodegeneration. In parallel, transcriptomic and functional studies of *APOE3*-Christchurch astrocytes demonstrate enhanced lysosomal degradation of internalized tau. Collectively, these mechanisms constrain tau amplification and preserve neuronal and synaptic integrity despite overwhelming amyloid pathology [7,32].

Therapeutic implications: *APOE3*-Christchurch provides a powerful proof-of-concept for tau-containment strategies that do not require amyloid removal. Therapeutic approaches inspired by this variant include blocking APOE–HSPG interactions using antibodies or small molecules, engineering Christchurch-mimetic APOE biologics, or modulating astrocytic lysosomal programs to enhance tau degradation. Targeting the APOE–extracellular matrix axis represents a precision resilience strategy aimed at arresting tau propagation and preserving cognition even in the presence of established amyloid pathology.

#### 3.1.3. APOE3-Jacksonville (V236E) (Tier B)

Normal function: Proper APOE folding and lipidation reduce pathological self-association, support cholesterol delivery to neurons, and modulate plaque-associated toxicity by shaping glial and neuritic responses to aggregated amyloid-β.

Resilience association: The rare APOE3-Jacksonville variant (V236E) has been associated with reduced dementia risk and favorable neuropathological features in sequencing-based genetic studies and functional analyses. Carriers exhibit attenuated neuritic dystrophy, lower tau pathology relative to amyloid burden, and preserved neuronal integrity compared with APOE3 or APOE4 backgrounds, consistent with a probable cognitive resilience phenotype rather than simple reduction in pathology [8].

Proposed mechanism: The V236E substitution lies within the C-terminal lipid-binding domain of APOE and enhances protein conformational stability while reducing pathological self-aggregation. This structural stabilization improves APOE lipidation and limits the formation of neurotoxic ApoE–amyloid assemblies that promote neuritic damage and microglial inflammatory activation. By reducing plaque-associated toxicity and preserving membrane repair capacity, APOE3-Jacksonville likely raises the threshold at which amyloid deposition triggers synaptic loss and downstream tau-mediated neurodegeneration. These effects mirror, in attenuated form, the protective properties observed with *APOE* ε2 while avoiding the deleterious aggregation-prone features of *APOE* ε4 [8,33].

Therapeutic implications: APOE3-Jacksonville supports therapeutic strategies aimed at stabilizing APOE structure, enhancing lipidation, or preventing pathological APOE aggregation. Pharmacologic or biologic interventions that shift APOE toward a Jacksonville-like functional state represent a rational resilience-mimicking strategy to protect cognition in the presence of amyloid pathology.

Table 3 summarizes key structural and functional differences among major APOE variants. Variants are compared based on amino acid substitutions, effects on lipid binding and receptor interactions, AD risk, and emerging evidence. Protective variants including *APOE* ε2, APOE3 Christchurch, and APOE3 Jacksonville are associated with enhanced lipid homeostasis, reduced tau propagation and the preservation of cognitive function despite neuropathological burden. In contrast, APOE4 confers increased AD risk via impaired lipid transport and accelerated synaptic dysfunction.

#### 3.1.4. *CLU* rs11136000 (Tier A)

Normal function: Clusterin (*CLU*) participates in lipid transport, binds misfolded and aggregation-prone proteins, and modulates inflammatory and apoptotic signaling. In the context of AD, *CLU* interacts directly with amyloid-β (Aβ), influences its solubility and aggregation state, and facilitates clearance pathways across the blood–brain barrier via receptor-mediated transport mechanisms.

Resilience association: The *CLU* rs11136000 protective allele has been repeatedly associated with reduced AD risk, delayed cognitive decline, and preserved cognitive performance across large genome-wide association studies and longitudinal aging cohorts spanning multiple ancestries. Importantly, *CLU*-associated protection extends beyond simple amyloid burden reduction and has been linked to resilience-relevant phenotypes, including better cognitive outcomes relative to neuropathological load [10,36].

Proposed mechanism: Clusterin functions as an extracellular molecular chaperone that binds soluble Aβ species, preventing toxic oligomer formation and promoting clearance rather than deposition. *CLU*–Aβ complexes are efficiently transported across the blood–brain barrier via LRP2/megalin-mediated transcytosis, reducing extracellular amyloid toxicity. Through combined effects on protein chaperoning, lipid homeostasis, and glial support, *CLU* raises the threshold at which amyloid pathology induces synaptic dysfunction and cognitive decline [36,37].

Therapeutic implications: *CLU* biology supports resilience-oriented therapeutic strategies aimed at enhancing extracellular chaperone capacity and clearance efficiency rather than directly targeting amyloid production. Approaches that increase *CLU* expression or mimic its chaperone function may reduce soluble Aβ toxicity, protect synapses, and preserve cognition even in the presence of established amyloid pathology, making *CLU* an attractive target for combination resilience therapies.

#### 3.1.5. *ATP8B1* rs2571244 (Tier B)

Normal function: *ATP8B1* encodes a P4-type ATPase phospholipid flippase that maintains membrane lipid asymmetry by translocating phosphatidylserine and phosphatidylethanolamine across cellular membranes. While best characterized in hepatocytes, *ATP8B1* plays a central role in bile acid transport, lipid homeostasis, and membrane stability—processes with systemic metabolic and vascular implications relevant to brain health.

Resilience association: Variants in *ATP8B1* have been identified in genome-wide analyses of cognitive resilience, particularly in studies modeling cognitive performance relative to amyloid burden. Integrative genetic and regulatory analyses link *ATP8B1* to resilience-relevant phenotypes rather than simple AD risk reduction, suggesting a modulatory role in buffering neurodegeneration under pathological stress [12,38].

Proposed mechanism: *ATP8B1*-associated resilience likely reflects indirect metabolic and vascular buffering rather than direct effects on amyloid or tau processing. Altered bile acid composition and signaling through nuclear and membrane receptors such as FXR and TGR5 can influence neuroinflammation, cerebral metabolism, and blood–brain barrier function. Neuroprotective bile acid profiles have been shown to reduce inflammatory signaling and improve mitochondrial and vascular resilience, thereby limiting neuronal vulnerability to amyloid- and tau-mediated stress. Through this liver–brain axis, *ATP8B1* variants may indirectly support cognitive resilience by stabilizing systemic metabolic and vascular environments that reduce neuronal vulnerability to amyloid- and tau-associated stress, although direct cognitive outcome associations remain limited.

Therapeutic implications: *ATP8B1* highlights bile acid signaling and systemic metabolic regulation as underexplored resilience pathways in Alzheimer’s disease. Therapeutic strategies targeting bile acid receptors or modifying bile acid composition—several of which are already clinically approved for hepatic disorders—may offer repurposing opportunities to enhance brain resilience and preserve cognition in individuals with established AD pathology.

#### 3.1.6. APP Icelandic (A673T) (Tier D—Pathology Resistance Comparator)

Normal function: Amyloid precursor protein (APP) undergoes sequential proteolytic processing via either the non-amyloidogenic α-secretase pathway or the amyloidogenic β- and γ-secretase pathway, the latter generating amyloid-β (Aβ) peptides that aggregate into plaques and initiate downstream neurodegenerative cascades. APP processing, therefore, sits at the apex of AD pathogenesis.

Resistance association: The rare APP Icelandic variant (A673T), located adjacent to the β-secretase (BACE1) cleavage site, is strongly associated with reduced lifetime risk of AD and age-related cognitive decline. Carriers demonstrate significantly lower amyloid burden and delayed or absent clinical dementia, even at advanced ages. Importantly, protection conferred by A673T reflects reduced pathology formation rather than preserved cognition in the presence of established pathology, distinguishing this variant as a resistance—not resilience—mechanism [9,39].

Proposed mechanism: The A673T substitution directly reduces the efficiency of BACE1-mediated cleavage of APP, resulting in decreased production of Aβ peptides, particularly the aggregation-prone Aβ42 species. In addition, the mutation reduces the aggregation propensity and neurotoxicity of any Aβ that is produced. By attenuating the initiating step of the amyloid cascade, APP A673T prevents downstream tau pathology, synaptic dysfunction, and neurodegeneration from occurring, thereby lowering disease risk upstream of resilience-relevant buffering mechanisms [9].

Therapeutic implications: APP Icelandic provides genetic validation of amyloid-lowering strategies as disease-preventive interventions but does not inform mechanisms of cognitive resilience once pathology is established. While BACE1 inhibition and APP-processing modulation remain relevant for primary prevention, they are unlikely to restore or preserve cognition in individuals with substantial amyloid and tau burden. Accordingly, APP A673T is included here as a conceptual comparator that highlights the distinction between pathology resistance and true cognitive resilience.

### 3.2. Synaptic Function and Neuroplasticity

Resilience may arise from preserving excitatory–inhibitory balance, maintaining synaptic architecture, and sustaining compensatory network responses despite amyloid/tau exposure.

#### 3.2.1. *RELN*-COLBOS (H3447R) (Tier A) (COLBOS Research Study)

Normal function: Reelin (RELN) is a large extracellular matrix glycoprotein that plays essential roles in neuronal migration during development and in synaptic plasticity and circuit stability in the adult brain. In mature neurons, Reelin binds the lipoprotein receptors ApoER2 and VLDLR, triggering phosphorylation of the intracellular adaptor Disabled-1 (Dab1) and activation of downstream signaling cascades, including PI3K–Akt. This pathway enhances NMDA receptor function, stabilizes long-term potentiation, and negatively regulates glycogen synthase kinase-3β (GSK3β), a principal kinase responsible for tau hyperphosphorylation. Through these actions, Reelin signaling directly links synaptic maintenance to tau homeostasis [40,41].

Resilience association: *RELN*-COLBOS (H3447R) was identified in a heterozygous state in a PSEN1 E280A mutation carrier from the Colombian kindred who remained cognitively unimpaired until approximately two decades beyond the expected age of symptom onset. Despite carrying a fully penetrant autosomal-dominant AD mutation and exhibiting substantial amyloid pathology, this individual demonstrated preserved cognition and markedly reduced tau burden in vulnerable cortical regions. This profound delay of clinical expression in the presence of pathogenic amyloid provides compelling human evidence for a synaptic and tau-modulating mechanism of cognitive resilience. Given the rarity of *RELN*-COLBOS, these findings are best viewed as hypothesis-generating rather than universally generalizable [7,42].

Proposed mechanism: The H3447R substitution is located in the C-terminal region of Reelin and confers a gain-of-function effect on Reelin signaling. Functional analyses indicate enhanced Dab1 phosphorylation and sustained activation of the PI3K–Akt pathway, leading to inhibitory phosphorylation of GSK3β and consequent suppression of tau hyperphosphorylation. In parallel, amplified Reelin signaling strengthens NMDA receptor–dependent synaptic transmission and preserves dendritic spine integrity, counteracting amyloid-induced synaptic depression. By simultaneously limiting tau pathology and stabilizing synaptic and network function, *RELN*-COLBOS uncouples amyloid accumulation from downstream neurodegeneration, maintaining circuit integrity in regions that are typically among the earliest affected in AD [40,42].

Therapeutic implications: *RELN*-COLBOS provides direct genetic validation for resilience-oriented strategies that enhance Reelin signaling or its downstream tau-suppressive pathways. Potential therapeutic approaches include Reelin supplementation, gene therapy to augment Reelin expression, or pharmacologic activation of Dab1–PI3K–Akt signaling to inhibit GSK3β-mediated tau phosphorylation.

#### 3.2.2. NPTX2 (Tier A; Biomarker-Linked)

Normal function: Neuronal pentraxin 2 (NPTX2; also known as NARP) is an activity-dependent secreted protein expressed primarily by excitatory pyramidal neurons. NPTX2 plays a critical role in maintaining excitatory–inhibitory (E/I) balance by clustering AMPA receptors, particularly GluA4-containing receptors, on parvalbumin-positive (PV+) fast-spiking interneurons. Through this mechanism, NPTX2 ensures adequate excitatory drive onto inhibitory circuits, supporting gamma-frequency oscillations that are essential for attention, memory encoding, and network stability.

Resilience association: Across multiple biomarker-informed and longitudinal cohort studies, higher cerebrospinal fluid (CSF) and cortical NPTX2 levels are consistently associated with preserved cognitive performance, slower cognitive decline, and reduced clinical expression of dementia relative to amyloid and tau burden. Importantly, NPTX2 levels decline early in AD and strongly predict progression from cognitively normal status to mild cognitive impairment, positioning NPTX2 as a functional marker of network resilience rather than a modifier of pathology accumulation. Individuals with maintained NPTX2 levels demonstrate cognitive outcomes that exceed expectations based on their pathological or biomarker profiles, fulfilling criteria for cognitive resilience [43].

Proposed mechanism: NPTX2-mediated stabilization of excitatory input to PV+ interneurons preserves inhibitory control over cortical networks, preventing the E/I imbalance, hypersynchrony, and network noise induced by amyloid-β and tau pathology. Loss of NPTX2 leads to impaired gamma oscillations, interneuron dysfunction, and circuit-level instability, which precede overt neurodegeneration. This mechanism represents a form of functional resilience operating at the network level rather than through direct modulation of protein aggregation [43,44].

Therapeutic implications: NPTX2 highlights synaptic and circuit stabilization as a viable resilience-based therapeutic strategy in AD. Potential approaches include activity-dependent synapse stabilization, interneuron-supportive therapies, or NPTX2-targeted replacement or augmentation strategies.

#### 3.2.3. NEDD9 rs760678 (Tier B)

Normal function: Neural precursor cell expressed, developmentally downregulated 9 (NEDD9), also known as HEF1 or CAS-L, is a scaffolding and adaptor protein involved in integrin signaling, cytoskeletal remodeling, and cellular stress responses. In neurons, NEDD9 localizes to dendritic compartments and the post-synaptic density, where it interacts with focal adhesion kinase (FAK), Src family kinases, and actin-regulatory proteins to support dendritic spine formation, synaptic stability, and activity-dependent structural plasticity.

Resilience association: Genetic variation in NEDD9, including the rs760678 polymorphism, has been associated with cognitive reserve and resilience-related phenotypes in aging and AD cohorts. While replication remains emerging, available human genetic and neuropathological studies suggest that NEDD9 variants may modulate susceptibility to synaptic loss and cognitive decline relative to pathological burden, supporting classification as a probable cognitive resilience factor rather than a primary risk or resistance gene [45].

Proposed mechanism: NEDD9-mediated scaffolding of the integrin–FAK–Src signaling pathways promotes actin cytoskeleton stability within dendritic spines, a structural feature closely linked to cognitive resilience. In the context of AD, amyloid-β oligomers and tau pathology induce spine retraction and synaptic collapse through cytoskeletal destabilization. By maintaining synaptic structure despite ongoing pathology, NEDD9 may preserve network connectivity and cognitive function without directly altering amyloid or tau accumulation [46].

Therapeutic implications: NEDD9 highlights cytoskeletal stabilization and synaptic adhesion signaling as potential resilience-enhancing targets. Although direct therapeutic modulation of NEDD9 has not yet been explored, pathways upstream and downstream of NEDD9—such as integrin signaling and actin-regulatory mechanisms—represent candidate intervention points for preserving synaptic integrity in neurodegenerative disease.

### 3.3. Endosomal–Lysosomal Function, Proteostasis, and Tau Regulation

Endocytosis, vesicular trafficking, lysosomal degradation, and autophagy are central to APP processing, Aβ clearance, tau turnover, and synaptic maintenance.

#### 3.3.1. *RAB10* rs142787485 (Tier A)

Normal function: *RAB10* is a small Rab GTPase that regulates intracellular vesicular trafficking, including endosomal recycling, Golgi-to-plasma membrane transport, and secretory pathway dynamics. In neurons, *RAB10* influences endosomal maturation and the trafficking of cargo relevant to amyloid precursor protein (APP) processing, synaptic vesicle turnover, and lysosomal positioning. *RAB10* is also a key substrate of the LRRK2 kinase, linking vesicular trafficking to neurodegenerative signaling pathways.

Resilience association: Rare loss-of-function or expression-reducing variants in *RAB10*, including rs142787485, have been associated with reduced AD risk and resilience-related phenotypes in large-scale human genetic studies. Protective *RAB10* variants correlate with favorable biomarker profiles, including reduced tau pathology relative to amyloid burden and slower cognitive decline, supporting classification as a definitive resilience locus rather than a simple risk modifier [12,47].

Proposed mechanism: Reduced *RAB10* activity shifts intracellular trafficking dynamics in ways that limit neurodegeneration despite ongoing pathology. First, decreased *RAB10* function enhances retromer-mediated recycling of APP away from late endosomes, where β-secretase cleavage occurs, thereby reducing amyloidogenic processing without fully suppressing APP expression. Second, *RAB10* regulates exocytic pathways implicated in tau secretion; reduced *RAB10* activity limits extracellular release and trans-neuronal spread of tau seeds. Third, attenuation of LRRK2-mediated *RAB10* phosphorylation preserves lysosomal integrity and prevents vesicular congestion that otherwise accelerates synaptic failure. Together, these effects uncouple amyloid accumulation from tau propagation and synaptic loss, preserving neuronal function under pathological stress [47,48].

Therapeutic implications: *RAB10* represents a high-value resilience target for therapeutic modulation. Partial inhibition of *RAB10* expression or activity—via antisense oligonucleotides, RNA interference, or upstream LRRK2 modulation—could recapitulate protective trafficking states while avoiding global suppression of vesicular transport.

#### 3.3.2. *PICALM* rs3851179 (Tier A)

Normal function: Phosphatidylinositol binding clathrin assembly protein (*PICALM*) is a central adaptor protein required for clathrin-mediated endocytosis. *PICALM* recruits clathrin and accessory proteins to the plasma membrane, facilitating vesicle formation and cargo internalization. In the brain, *PICALM* is expressed in neurons and is highly enriched in cerebrovascular endothelial cells, where it plays a critical role in blood–brain barrier (BBB) transcytosis and clearance pathways.

Resilience association: The *PICALM* rs3851179 protective allele is one of the most consistently replicated genetic modifiers associated with reduced AD risk across diverse populations. Beyond risk reduction, *PICALM* variants are linked to resilience-relevant biomarker profiles, including lower brain amyloid burden, preserved cognitive function relative to pathology, and delayed clinical progression, supporting its classification as a Tier A resilience gene [49].

Proposed mechanism: *PICALM* promotes cognitive resilience by enhancing amyloid clearance and maintaining endosomal homeostasis. In cerebrovascular endothelial cells, *PICALM* binds to LRP1–Aβ complexes and facilitates their internalization and transcytosis across the BBB into the circulation, constituting a major route of amyloid clearance from the brain. In neurons, *PICALM* regulates synaptic vesicle recycling and autophagic trafficking, preventing endosomal overload and preserving synaptic transmission under amyloid stress. By strengthening vascular and neuronal clearance mechanisms, *PICALM* limits the accumulation of toxic Aβ species and indirectly reduces downstream tau pathology and synaptic dysfunction [50].

Therapeutic implications: *PICALM* biology motivates resilience-based therapeutic strategies aimed at enhancing BBB-mediated clearance and endosomal trafficking efficiency. Interventions that increase endothelial *PICALM* expression or function may augment amyloid efflux from the brain, while neuronal *PICALM* modulation could preserve synaptic resilience. These approaches are particularly attractive as complements to amyloid- or tau-targeted therapies.

#### 3.3.3. CTSH rs2289702 (Tier B)

Normal function: Cathepsin H (CTSH) is a lysosomal cysteine protease expressed in neurons and glial cells, particularly microglia. CTSH contributes to proteolytic degradation of internalized proteins within the lysosome and plays an important role in phagolysosomal processing of amyloid-β and cellular debris.

Resilience association: Protective regulatory variants in CTSH, including rs2289702, have been associated with reduced AD risk and resilience-linked biomarker signatures in human genetic studies. CTSH expression levels correlate with microglial clearance capacity and cognitive outcomes relative to pathological burden, supporting its classification as a probable resilience factor [51,52].

Proposed mechanism: Increased CTSH expression or activity enhances lysosomal proteolytic capacity within microglia, improving degradation of phagocytosed amyloid fibrils and preventing accumulation of indigestible lysosomal inclusions that drive microglial senescence. By optimizing proteostasis rather than suppressing amyloid production, CTSH promotes resilience to pathology-induced neurodegeneration [53].

Therapeutic implications: CTSH highlights lysosomal tuning as a resilience-enhancing strategy distinct from direct amyloid targeting. Pharmacologic or genetic approaches that boost lysosomal protease activity in microglia may improve clearance efficiency and delay neurodegeneration without exacerbating inflammation, offering a complementary avenue for combination therapy.

#### 3.3.4. TFEB Axis (Tier B; Pathway-Level)

Normal function: Transcription factor EB (TFEB) is the master regulator of the autophagy–lysosomal pathway, coordinating expression of genes involved in lysosomal biogenesis, autophagosome formation, and intracellular trafficking. TFEB activation enhances cellular capacity to degrade misfolded proteins, damaged organelles, and aggregated substrates, thereby maintaining proteostasis.

Resilience association: Convergent evidence from human transcriptomic analyses, biomarker-informed cohorts, and experimental models links enhanced autophagy–lysosomal programs with preserved cognitive function and slower neurodegeneration in AD. While no single TFEB coding variant has been definitively associated with resilience, sustained TFEB pathway activity is a recurring molecular signature of resilience-associated states [54,55].

Proposed mechanism: Activation of the TFEB axis increases lysosomal number and function, facilitating the clearance of amyloid-β, hyperphosphorylated tau, and damaged mitochondria. Enhanced autophagic flux also supports synaptic maintenance and metabolic resilience, buffering neurons against cumulative stress [56,57,58].

Therapeutic implications: The TFEB pathway represents a promising resilience-mimicking target for pharmacologic intervention. Small molecules that promote TFEB nuclear translocation or autophagy activation may enhance proteostasis and delay cognitive decline when applied alone or in combination with amyloid- or tau-directed therapies. Importantly, TFEB modulation offers a pathway-level approach that does not rely on targeting a single pathogenic protein.

### 3.4. Neuroinflammation and Immune Response

Microglial state transitions, inflammatory thresholds, and innate immune signaling can promote clearance and repair or drive synaptic injury, making immune tuning a major resilience axis.

#### 3.4.1. *PLCG2* P522R (Tier A)

Normal function: Phospholipase C gamma 2 (*PLCG2*) is a signaling enzyme expressed predominantly in myeloid cells, including microglia in the central nervous system. *PLCG2* hydrolyzes phosphatidylinositol-4,5-bisphosphate (PIP2) to generate inositol trisphosphate (IP3) and diacylglycerol (DAG), initiating calcium-dependent signaling cascades that regulate microglial activation, survival, phagocytosis, and inflammatory responses.

Resilience association: The rare missense variant *PLCG2* P522R is one of the most robustly replicated protective variants in AD genetics. Across large genome-wide association studies and follow-up functional analyses, P522R is consistently associated with reduced AD risk, delayed cognitive decline, and favorable biomarker trajectories. Importantly, the protective effect of *PLCG2* P522R appears to operate downstream of amyloid deposition, implicating immune resilience rather than primary resistance to pathology formation [59,60].

Proposed mechanism: The P522R substitution confers a mild gain-of-function effect that enhances *PLCG2* enzymatic activity without triggering excessive inflammatory signaling. This tuned activation promotes microglial phagocytosis, lipid handling, and survival while limiting chronic pro-inflammatory cytokine release. By optimizing microglial responsiveness rather than suppressing immune activity, *PLCG2* P522R establishes a protective activation state that buffers neural circuits from pathology-induced damage [61,62].

Therapeutic implications: *PLCG2* provides compelling genetic validation for microglial tuning as a resilience-based therapeutic strategy. Pharmacologic approaches that partially enhance *PLCG2* signaling—or selectively activate downstream pathways that promote phagocytosis and repair—may recapitulate the protective immune states observed in P522R carriers while avoiding the risks associated with broad immune suppression or overactivation.

#### 3.4.2. SPI1/PU.1 rs1057233 (Tier B)

Normal function: SPI1 encodes PU.1, a transcription factor that serves as a master regulator of myeloid lineage identity and function. In microglia, PU.1 controls broad transcriptional programs governing immune surveillance, inflammatory responsiveness, phagocytosis, and metabolic state. PU.1 expression levels strongly influence microglial phenotype and reactivity.

Resilience association: Genetic variants associated with reduced SPI1 expression, including rs1057233, are linked to delayed Alzheimer’s disease onset and reduced disease risk in human genetic studies. Lower PU.1 activity correlates with attenuated microglial inflammatory responses and improved cognitive outcomes relative to pathological burden, supporting its classification as a probable resilience modifier rather than a direct pathology-reducing factor [21,63,64].

Proposed mechanism: Reduced PU.1 expression shifts microglial transcriptional programs away from pro-inflammatory and neurotoxic states toward phenotypes characterized by improved phagocytic efficiency and reduced cytokine-mediated synaptic injury. This transcriptional rebalancing allows microglia to support neuronal resilience rather than amplify neurodegeneration.

Therapeutic implications: SPI1 highlights transcriptional-state modulation as a potential resilience strategy in AD. Partial inhibition of PU.1 activity or downstream inflammatory gene programs could promote protective microglial states without abolishing innate immune function. Such approaches may require careful titration to preserve clearance capacity while minimizing inflammatory toxicity.

#### 3.4.3. IL1RL1 rs1921622 (Tier B)

Normal function: IL1RL1 encodes ST2, a receptor for the cytokine interleukin-33 (IL-33). The IL-33/ST2 signaling axis regulates innate immune activation, microglial polarization, and inflammatory balance within the central nervous system.

Resilience association: Protective variants in IL1RL1, including rs1921622, have been associated with reduced AD risk and resilience-linked phenotypes in human cohorts. These effects show modulation by sex and ancestry, suggesting context-dependent immune regulation rather than uniform protection across populations [65,66,67].

Proposed mechanism: Reduced expression of soluble ST2 enhances IL-33 signaling through membrane-bound IL1RL1 on microglia. IL-33 activation promotes microglial phagocytosis, supports synaptic maintenance, and limits chronic inflammatory signaling. By sustaining IL-33-mediated repair programs, IL1RL1 protective variants may enhance clearance of pathological substrates and preserve neuronal function without exacerbating neuroinflammation [68,69].

Therapeutic implications: The IL-33/ST2 axis represents a precision immune resilience pathway that may benefit from sex- or ancestry-stratified therapeutic approaches. Enhancing IL-33 signaling or reducing soluble ST2 levels could promote protective microglial responses, although careful dosing will be essential to avoid excessive immune activation.

#### 3.4.4. TREM2 (Tier B; Context-Dependent)

Normal function: Triggering receptor expressed on myeloid cells 2 (TREM2) is a lipid-sensing receptor expressed by microglia that regulates cell survival, metabolic fitness, chemotaxis, and response to amyloid plaques. TREM2 signaling enables microglia to cluster around plaques, restrict their expansion, and adopt disease-associated microglial (DAM) states.

Resilience association: TREM2 variants exhibit context-dependent effects on AD risk and progression. While loss-of-function variants such as R47H increase AD risk, intact or appropriately activated TREM2 signaling has been associated with improved plaque containment, reduced neuritic damage, and preserved cognition under certain pathological conditions. These divergent effects underscore the importance of signaling balance rather than absolute activation [70,71].

Proposed mechanism: Properly tuned TREM2 signaling promotes microglial survival and plaque encapsulation, limiting diffusion of toxic amyloid species and reducing bystander synaptic injury. Thus, TREM2 contributes to resilience when activation thresholds are optimized to support clearance and containment without triggering chronic neuroinflammation [72,73].

Therapeutic implications: TREM2 biology supports carefully calibrated microglial receptor agonism as a resilience strategy. Therapeutic efforts should aim to restore physiologic TREM2 signaling levels rather than indiscriminate activation, emphasizing timing, disease stage, and patient genotype to achieve beneficial outcomes.

#### 3.4.5. PLA2G4A (Tier C)

Normal function: PLA2G4A encodes cytosolic phospholipase A2 group IVA (cPLA2), an enzyme that catalyzes the release of arachidonic acid from membrane phospholipids. This reaction generates bioactive lipid mediators, including prostaglandins and leukotrienes, which regulate synaptic signaling, inflammation, and neuronal excitability.

Resilience association: Although direct human genetic evidence for clinicopathologic dissociation is limited, experimental and translational studies demonstrate that genetic or pharmacologic reduction of cPLA2 activity preserves cognitive function in the presence of substantial amyloid burden. These findings support classification of PLA2G4A as a putative resilience-related pathway rather than a primary genetic resilience locus [74]. Notably, direct human genetic evidence linking PLA2G4A variants to clinicopathologic dissociation remains limited, and current support is largely derived from experimental and translational studies.

Proposed mechanism: Amyloid-β oligomers aberrantly activate cPLA2, leading to excessive arachidonic acid release and downstream production of pro-inflammatory lipid mediators that disrupt synaptic transmission and plasticity. Inhibition of cPLA2 attenuates amyloid-induced synaptic dysfunction and excitotoxicity without altering amyloid deposition, indicating that cPLA2 acts downstream of pathology to mediate neurotoxicity. This decoupling of pathology from functional impairment represents a mechanistic form of resilience at the signaling level [75].

Therapeutic implications: PLA2G4A highlights lipid-mediated neuroinflammatory signaling as a modifiable resilience pathway. Targeting cPLA2 or its downstream eicosanoid pathways may preserve synaptic function and cognition even after amyloid pathology is established, offering potential adjunctive therapies for later disease stages.

### 3.5. Vascular/BBB Integrity and Extracellular Matrix Mechanisms

Cognitive resilience can arise from preservation of cerebrovascular integrity, efficient blood–brain barrier (BBB) transport, and extracellular matrix (ECM) states that support clearance while limiting chronic gliosis. Vascular dysfunction and BBB breakdown are increasingly recognized as early and independent contributors to cognitive decline in AD, amplifying neuroinflammation, impairing metabolic support, and restricting the removal of toxic proteins. Genetic variants that stabilize vascular–ECM interactions and BBB function may therefore decouple neuropathology from neurodegeneration.

#### *FN1* rs140926439 (Tier A)

Normal function: Fibronectin (*FN1*) is a major extracellular matrix glycoprotein involved in cell adhesion, integrin signaling, and structural organization of the vascular basement membrane. Within the neurovascular unit, *FN1* contributes to BBB integrity, regulates endothelial–pericyte interactions, and influences leukocyte trafficking and inflammatory signaling. *FN1* deposition is dynamically regulated in response to injury and inflammation.

Resilience association: Rare protective variation in *FN1*, including rs140926439, has been associated with preserved cognitive outcomes and reduced AD risk in large-scale human genetic analyses. Notably, *FN1*-associated protection is enriched among high-risk *APOE* ε4 carriers, suggesting a modifier role that buffers vulnerability rather than preventing pathology formation outright. These findings support *FN1* as a definitive resilience gene acting through vascular and extracellular mechanisms [76].

Proposed mechanism: Excessive fibronectin accumulation at the BBB is a hallmark of cerebrovascular pathology and contributes to endothelial dysfunction, perivascular gliosis, and impaired clearance of amyloid-β. Protective *FN1* variants are hypothesized to limit pathological ECM deposition, preserving BBB permeability and facilitating transvascular clearance pathways mediated by LRP1 and related transporters. Therapeutic implications: *FN1* highlights ECM remodeling and BBB stabilization as underexplored resilience-based therapeutic axes. Interventions that normalize vascular ECM composition, reduce fibronectin overaccumulation, or strengthen neurovascular unit function may synergize with amyloid- and tau-directed therapies by restoring clearance capacity and limiting secondary inflammatory injury. Such strategies may be particularly beneficial in genetically high-risk populations.

### 3.6. RNA Regulation and Systems-Level Resilience (Emerging)

RNA-binding and post-transcriptional regulation can coordinate multi-pathway resilience, particularly in immune cells and synaptic maintenance programs.

#### MicroRNA-Related Regulatory Variants (Tier C)

Normal function: MicroRNAs are small non-coding RNAs that regulate gene expression by binding complementary sequences in target mRNAs, leading to translational repression or transcript degradation. In the brain, miRNAs orchestrate activity-dependent synaptic plasticity, inflammatory responses, and stress adaptation by coordinating networks of genes rather than single molecular targets.

Resilience association: Genetic variants affecting miRNA genes or miRNA-binding sites within target transcripts have been linked to AD risk and altered expression of resilience-relevant pathways. While direct evidence for clinicopathologic dissociation remains limited, several miRNA signatures correlate with preserved cognition, reduced synaptic loss, or attenuated neuroinflammatory responses relative to pathological burden, suggesting a modulatory role in disease expression [77,78].

Proposed mechanism: miRNA-mediated regulation enables coordinated suppression or enhancement of multiple components within amyloid, tau, inflammatory, and synaptic pathways. By dampening pro-inflammatory signaling, stabilizing synaptic gene networks, and supporting proteostatic capacity, protective miRNA profiles may raise the threshold at which pathological insults translate into functional impairment. This systems-level buffering distinguishes RNA-based resilience mechanisms from single-gene effects and may explain interindividual variability in disease progression.

Although still emerging, RNA-based resilience mechanisms suggest potential network-level intervention strategies. Modulating specific miRNAs or miRNAs–mRNA interactions could theoretically reinforce protective transcriptional programs across multiple pathways simultaneously. However, substantial challenges remain, including delivery, specificity, and the need for stronger human genetic and longitudinal validation before clinical translation.

## 4. Discussion

This review highlights cognitive resilience as a distinct and underappreciated dimension of AD biology in which preserved cognitive function can occur despite substantial amyloid and tau pathology. Rather than reflecting a failure of pathological accumulation, resilience represents an active biological state in which neural systems buffer, compensate for, or tolerate proteotoxic stress. Across human genetic studies and extreme resilience of phenotypes, resilience-associated variants converge on a limited set of interacting biological mechanisms that collectively decouple neuropathology from clinical dementia.

Specifically, resilience genes converge on pathways that (i) limit tau propagation and downstream neurodegeneration, (ii) preserve synaptic integrity and excitatory–inhibitory network balance, (iii) optimize endosomal–lysosomal trafficking and proteostatic capacity, (iv) tune microglial activation thresholds toward reparative rather than neurotoxic states, and (v) maintain vascular and blood–brain barrier (BBB) integrity alongside extracellular matrix (ECM) conditions that support clearance and minimize gliosis. Taken together, these findings indicate that cognitive resilience is not mediated by a single protective mechanism but instead reflects a multi-system buffering program operating across neuronal, glial, vascular, and systemic domains.

While resilience-associated pathways offer promising therapeutic targets, translating these mechanisms into safe and effective interventions presents substantial challenges. Many resilience mechanisms operate within narrow functional windows, where insufficient activation fails to confer benefit and excessive activation risks toxicity or immune overstimulation. In addition, gene delivery, blood–brain barrier penetration, cell-type specificity, and disease-stage timing remain major barriers for resilience-mimicking therapies. These constraints underscore the need for precision approaches that modulate resilience pathways in a controlled and context-dependent manner rather than broad pathway activation.

### 4.1. Convergent Mechanistic Themes

Several unifying themes emerge from the genetic architecture of cognitive resilience. First, variants within the APOE–lipid axis illustrate that lipid metabolism and extracellular interactions exert a powerful influence on the coupling between amyloid burden and tau-driven neurodegeneration. Protective APOE variants and lipid-associated chaperones such as *CLU* modify membrane dynamics, aggregation propensity, and clearance efficiency, thereby reducing the neurotoxic consequences of amyloid without necessarily preventing its accumulation.

Second, synapse- and circuit-level mechanisms—including Reelin signaling, NPTX2-mediated excitatory–inhibitory balance, and cytoskeletal stabilization via NEDD9—demonstrate that preservation of network function can substantially delay clinical expression even in the presence of established pathology. These findings reinforce the concept that synaptic and network integrity represents proximate determinants of cognition and is therefore critical targets for resilience-based intervention.

Third, endosomal–lysosomal and vesicular trafficking pathways (e.g., *RAB10*, *PICALM*, CTSH, and TFEB-associated programs) define a mechanistic axis through which intracellular clearance capacity and proteostasis can be enhanced. By limiting endosomal congestion, facilitating amyloid and tau clearance, and preventing proteostatic collapse, these pathways reduce downstream synaptic injury and neuronal vulnerability.

Finally, immune-related variants (*PLCG2*, SPI1/PU.1, IL1RL1, and context-dependent TREM2 signaling) emphasize that microglial states are not uniformly pathogenic. Instead, microglial activation exists along a spectrum in which appropriately tuned signaling promotes clearance, tissue repair, and synaptic preservation, whereas maladaptive inflammatory activation accelerates neurodegeneration. Genetic resilience factors appear to shift microglial thresholds toward protective, metabolically competent states that support neuronal survival.

Importantly, vascular and BBB-associated mechanisms—exemplified by *FN1* and *PICALM*—integrate with immune and clearance pathways by preserving perfusion, limiting ECM-driven gliosis, and maintaining efficient transport routes for toxic protein removal. These observations underscore the central role of the neurovascular unit in resilience and highlight vascular health as an essential, genetically modulated component of cognitive preservation.

### 4.2. Therapeutic Implications

The genetic architecture of cognitive resilience motivates a shift in therapeutic strategy from exclusively targeting pathological aggregates toward preserving cognitive function by reinforcing endogenous buffering mechanisms. Rather than replacing disease-modifying approaches, resilience-based interventions are likely to be most effective when deployed in combination with amyloid- or tau-directed therapies.

Candidate resilience-oriented strategies include:

(1) Gene-based or gene-modulating interventions, aimed at introducing protective allelic effects (e.g., APOE2-like biology) or attenuating vulnerability pathways (e.g., partial suppression of *RAB10*-mediated trafficking programs).
(2) Microglial tuning therapies, targeting signaling nodes such as the *PLCG2*–TREM2 axis, IL-33/ST2 signaling, or transcriptional regulators including PU.1, with the goal of promoting reparative, clearance-competent immune states while avoiding chronic inflammatory activation.
(3) Proteostasis and autophagy enhancement, through pharmacologic activation of lysosomal and autophagic programs coordinated by TFEB and related pathways, thereby increasing cellular tolerance to accumulated misfolded proteins.
(4) Vascular and BBB-focused strategies, designed to strengthen clearance routes, normalize ECM composition, and preserve neurovascular unit integrity, as informed by *PICALM*- and *FN1*-associated resilience mechanisms.

Given that resilience mechanisms operate across interacting biological systems, the most effective future interventions are likely to involve precision combinations guided by genetic background, biomarker profiles, and disease stage, rather than single-target approaches.

**Figure 1:**
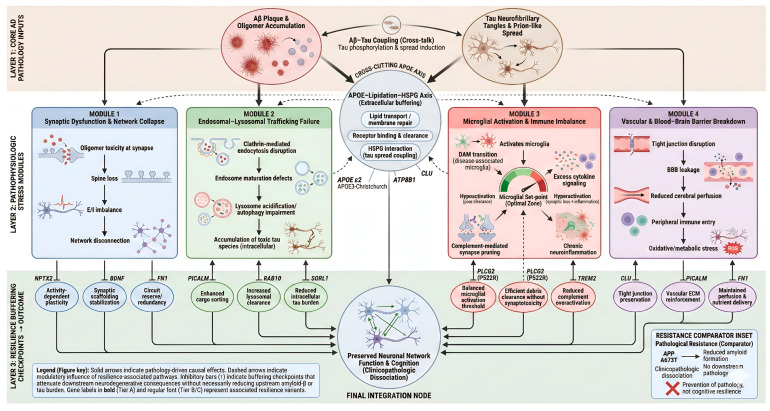
Systems-level pathway model of cognitive resilience to Alzheimer’s disease. This figure is an original illustration created by the authors using AI-assisted graphical rendering and does not reproduce or adapt previously copyrighted material.

The schematic diagram in Figure 1 illustrates a systems-level pathway model of cognitive resilience to Alzheimer’s disease at 4 module levels, including synaptic dysfunction and network collapse (module 1), endosomal-lysosomal trafficking failure (module 2), microglial activation and immune imbalance (module 3), and vascular and blood–brain barrier breakdown (module 4). The illustration also depicts AD pathophysiology and resilience at 3 layers. Layer 1 is the core hallmark features of AD, layer 2 is the pathophysiological processes that lead to neuronal dysfunction, and layer 3 is the final integration node that leads to preserved neuronal network function and cognition.

### 4.3. Limitations and Future Directions

Despite growing interest in cognitive resilience, current evidence remains constrained by several limitations. Extreme protective variants are rare, many studies exhibit ancestry imbalance, and resilience phenotypes are defined heterogeneously across cohorts. Moreover, integration of genetics with longitudinal biomarker data, multi-omics profiling, and functional validation remains incomplete.

Future research should prioritize standardized definitions of resilience, replication in diverse populations, and experimental systems, such as iPSC-derived neurons and glia, organoids, and humanized knock-in models, that directly test how protective variants uncouple pathology from neurodegeneration. Biomarker-informed clinical trials incorporating resilience-relevant endpoints, including network function, synaptic markers, and rates of neurodegeneration, will be essential for translating genetic insights into cognition-preserving therapies.

## 5. Conclusions

Protective genetic variants provide a powerful blueprint for understanding cognitive resilience in AD. Regarding lipid metabolism, synaptic and network maintenance, proteostasis, immune regulation, and vascular–BBB integrity, resilience genes converge on mechanisms that preserve neural function despite substantial pathological stress. Leveraging these naturally occurring protective strategies may enable a paradigm shift toward resilience-based precision interventions aimed not solely at reducing pathology, but at preventing or delaying dementia by strengthening the brain’s intrinsic capacity to tolerate disease.

## Figures and Tables

**Table 1:** Tier D genes are included solely as conceptual comparators. These variants reduce AD risk by preventing or attenuating pathology formation and therefore do not represent cognitive resilience, which requires preserved cognition despite substantial pathology.

Tier	Classification	Primary Phenotype Used for Ranking	Human Evidence Required	Mechanistic Requirement	How Genes Were Assigned
Tier A	Definitive Cognitive Resilience Genes	Preserved cognition despite moderate–high amyloid and/or tau pathology (clinicopathologic dissociation)	Replicated in multiple human cohorts or extreme resilience cases	Strong, pathway-consistent mechanisms validated experimentally	Preserved cognition despite moderate–high amyloid and/or tau pathology (clinicopathologic dissociation)
Tier B	Probable Cognitive Resilience Genes	Improved cognitive trajectory relative to pathological burden	Significant association in at least one well-powered human cohort	Partial or emerging mechanistic support	Genes with clear human relevance but incomplete replication or mechanistic depth
Tier C	Putative/Exploratory Resilience Candidates	Resilience inferred indirectly (biomarkers, network preservation, experimental models)	Limited or indirect human evidence	Strong mechanistic plausibility based on experimental data	Genes prioritized for biological relevance but lacking direct human resilience confirmation
Tier D	Pathology Resistance Genes (Comparator Category)	Reduced or delayed amyloid/tau pathology	Strong and replicated human genetic evidence	Clear mechanism limiting pathology formation	Genes excluded from resilience tiers because cognition is preserved by avoiding pathology

**Table 2:** Summary of resilience genes/variants, pathway, phenotype, mechanism, tier, references.

Gene	Variant	Pathway/Function	Resilience Trait	Mechanism	Evidence Tier	Representative Studies
Lipid Transport & Metabolism						
*APOE* ε2	rs7412 (Arg158Cys)	Lipid metabolism/AB clearance	Preserved cognition despite high amyloid load	Improves lipid efflux, synaptic repair, and microglial regulation	A	[6]
*APOE3* Christchurch	R136S	Lipid binding/tau propagation	Normal cognition until the 70s, exceptional cognitive resilience in a PSEN1 carrier	Reduces APOE-HSPG binding, impaired tau propagation	A	[7]
*APOE3* Jacksonville	V236E	Lipid transport/tau modulation	Preserved cognitive function, reduced tau deposition	Stabilized lipid dynamics, less neuronal stress	B	[8]
*APP* Icelandic	A673T	Amyloid processing	Reduced/resistance to AD incidence	Decreased Aβ production via β-secretase cleavage of APP	D	[9]
*CLU*	rs11136000	Lipid transport/chaperone-mediated clearance	Preserved cognitive resilience in aging	Facilitates AB transport via LRP1-endosomal pathway	A	[10]
*ATP8B1*	rs113985933	Lipid transport/phospholipid homeostasis	High cognitive resilience score/index	Reduced oxidative stress and lipid peroxidation	B	[11]
Synaptic Resilience & Plasticity						
*RELN*	H3447R	Synaptic signaling/Reelin-Dab1 pathway	Preserved memory performance despite amyloid burden	Reelin–Dab1–NMDA receptor stabilization	A	[12]
*NPTX2*	No specific human resilience variants	Synaptic homeostasis	Cognitive resilience index (preserved cognition despite AD pathology)	Promotes excitatory synaptic stabilization and neuronal plasticity; regulates AMPA receptor clustering	A	[13]
*NEDD9*	rs760678	Cytoskeletal dynamics/adhesion	Cognitive reserve in aging brains	Stabilizes dendritic spines and cytoskeletal stability	B	[14]
Cellular Trafficking & Autophagy						
*PICALM*	rs3851179	Endocytosis/vesicle trafficking	Memory resilience	Improves clathrin-mediated AB clearance and synaptic function	A	[15]
*TFEB*	No specific human resilience variants	Lysosomal-autophagy regulation	preserved cognition, reduced tau burden	Enhances lysosomal degradation of tau and Aβ	A	[16]
*CTSH*	rs2289702	Lysosomal protease	high resilience gene expression signature	Promotes proteostasis, reduces aggregates	B	[17]
*RAB10*	rs142787485	Vesicle transport/endosomal sorting	high cognitive resilience score	Reduces tau trafficking and synaptic loss	A	[18]
Immune Response & Microglial Regulation						
*PLCG2*	P522R	Immune signaling/microglial activation	Delayed cognitive decline	Enhances microglial phagocytosis, PI3K–AKT pathway activation	A	[19]
*TREM2*	R47H, R62H	Microglial lipid metabolism	Preserved cognition under mild pathology	Promotes Aβ phagocytosis	B	[20]
*SPI1* (PU.1)	rs1057233	Transcriptional control of immune genes	Delayed AD onset, preserved cognition	Reduces proinflammatory microglial activity	B	[21]
*IL1RL1*	rs1420101	Cytokine signaling	Enhanced microglial resilience marker	Reduces Aβ-driven inflammation	B	[22]
*PLA2G4E*	rs7694493	Lipid metabolism/arachidonic acid signaling	Preserved cognition with high amyloid load	Modulates arachidonic acid pathway, reduces neuroinflammation	C	[23]
Structural & ECM Integrity						
*FN1*	rs1046706	ECM and integrin signaling	Preserved cognition despite pathology	Integrin signaling reduces neurotoxicity	A	[24]

**Table 3:** APOE variants associated with cognitive resilience and AD risk.

APOE Variant	Structural Change	Functional Impact	AD Risk	Cognitive Resilience Evidence	Mechanistic Insight	Key Reference
APOE ε2	Arg158Cys substitution	Reduced LDLR binding, improved lipid transport	↓	Strong population-level resilience	Decreased Aβ aggregation and inflammation	[34]
APOE3 (wild-type)	None	Neutral lipid metabolism	0	Baseline reference	Normal lipid and receptor binding	[26]
APOE3 Christchurch (R136S)	Arg → Ser in receptor binding region	Reduced heparin binding affinity	↓↓	Very strong (Colombian PSEN1 case)	Impaired tau propagation, preserved neurons	[7]
APOE3 Jacksonville (V236E)	Arg → Ser in lipid-interaction domain	Altered lipid dynamics	↓	Emerging	Stabilizes membranes and reduces tauopathy	[8]
APOE4	Arg112Arg158 conformation	Domain destabilization, increased aggregation	↑↑	None	Promotes neuroinflammation and synaptic loss	[35]

## Data Availability

No new data were created or analyzed in this study. Data sharing does not apply to this article.
